# Development of an easy and cost-effective method for non-invasive genotyping of insects

**DOI:** 10.1371/journal.pone.0216998

**Published:** 2019-06-03

**Authors:** Bahar Ali, Yicheng Zhou, Qiuyuan Zhang, Changying Niu, Zhihui Zhu

**Affiliations:** Hubei Insect Resources Utilization and Sustainable Pest Management Key Laboratory, College of Plant Science & Technology, Huazhong Agricultural University, Wuhan, Hubei, China; University of Helsinki, FINLAND

## Abstract

Non-invasive genotyping methods provide valuable information on insect populations. However, poor DNA amplification and time-consuming sampling procedures limit these methods, especially for small insects. An efficient and convenient method was developed for non-invasive, non-lethal genotyping of a large insect, *Mythimna separata*, and a small insect, *Drosophila melanogaster*, by amplification of endogenous and exogenous, nuclear and mitochondrial genes from insect frass, exuviae, and food waste. For *M*. *separata*, the chitin synthesis gene *MsCHSB* and the *COI* gene were successfully detected by PCR from exuviae DNA. However, a *COI* fragment could not be detected directly by PCR from frass, probably due to DNA degradation. To improve the detection rate, DNA from frass was first amplified by Multiple Displacement Amplification with phi29 DNA polymerase, after which the *COI* fragment was detected from all samples by PCR. For *D*. *melanogaster*, second instar larvae were reared individually for three days and then DNA was extracted from food waste of each individual. The endogenous fragment *serendipity* α (sryα), exogenous transgene ΦC31 integrase, and the *kl-5* gene, a Y-chromosome-located male-specific marker gene were successfully detected from most samples. We developed a simple, non-invasive, non-lethal method to determine gender and identify transgenic individuals early in the larval stage. This universal method is applicable to most insects and has potential application in genetic and ecological studies of insects and other arthropods.

## Introduction

Molecular genetic methods, including DNA sequencing, are used routinely to maintain insect cultures and to carry out research into populations for development purposes. However, these methods usually result in the death of sampled insects, and this can be problematic in small captive populations where individuals are important for experimental purposes. [1]. In such cases, sampling of shed setae, feces or exuviae are preferable because their collection does not require capturing or harming the animals. In a study of predation of ground-nesting birds by wild boars, genetic analysis of wild boar scats revealed that predation was about five-times greater than that predicted by morphological examination. Moreover, this method allowed considerably more precise taxonomic identification of bird species compared with morphological analysis [2]. Increasingly, biologists depend on genetic analysis to deduce patterns of distribution, predict reproductive fitness, and evaluate long-term population feasibility in threatened species. For small creatures such as insects, the development of tissue sampling methods that do not cause individual mortality is a particular challenge because very small samples sizes may not provide sufficient DNA for genetic studies [3].

A single tibia sampled from live damselflies delivered sufficient tissue for DNA extraction without killing the insect [4]. Another non-lethal tissue source is hemolymph, as shown in studied with scorpion fly larvae and adults, although the effect of sampling on adult mortality rates was not reported [3,5]. Sufficient amounts of DNA were extracted by non-lethal sampling of 2 mm^2^ of wing edge [6], or 3 mm^2^ of wing tip [7] from butterflies. However, the clipping method may be unsuitable in some cases (e.g. museum specimens) or in cases of species that are more dependent on those body parts responsible for the reproduction, feeding, or movement.

Non-lethal techniques have been developed to extract insect DNA from frass (feces) and exuviae (shed exoskeleton) [8,9]. This approach is particularly useful for insects at the larval stage of development, where large quantities of frass are generated, or when instars shed their exoskeletons [10,11]. Before using an organism in a bioassay to distinguish between cryptic species, to differentiate cryptic morphs, or assess relatedness between organisms before a behavioral study, DNA must be obtained without affecting the fitness or behavior of the organism [8]. However, the detection rate is not high with these methods due to degradation of DNA by nucleases in frass or exuviae samples collected in the field [12]. Moreover, it is also time-consuming to collect enough fresh frass or exuviae samples from insects reared in a laboratory, and sometimes it is hard to collect these samples from small insects such as the common fruit fly, *Drosophila melanogaster*, and from the first instar larvae of many insects.

Oriental army worm, *Mythimna separata*, is one of the most damaging seasonal pests of cereal crops in Asia, especially in China. During their multi-generational, round-trip migration from southern to northern China, these insects damage thirty-three crop species in eight plant families [13–15]. Although migration is a characteristic of *M*. *separata*, not all individuals in a population show the same migratory behavior, and this may have a genetic basis. To identify the factors influencing migration behavior, it is essential to study individual genotypes. *Drosophila melanogaster* is the most well-studied model insect. Genetic manipulation in this species is routine, and so this specie is used as a model for expressing transgenes and for genome editing [16,17]. To the best of our knowledge, no non-lethal genotyping method is available for transgenic fruit fly identification, and for sex determination, mainly due to the small size of this organism. In this study, we aimed to develop a simple and simple and sensitive method for non-invasive, non-lethal genotyping of a large insect, *M*. *separata*, and a small insect, *D*. *melanogaster*, from frass, exuviae, and food waste samples.

## Results

**Fig 1:**
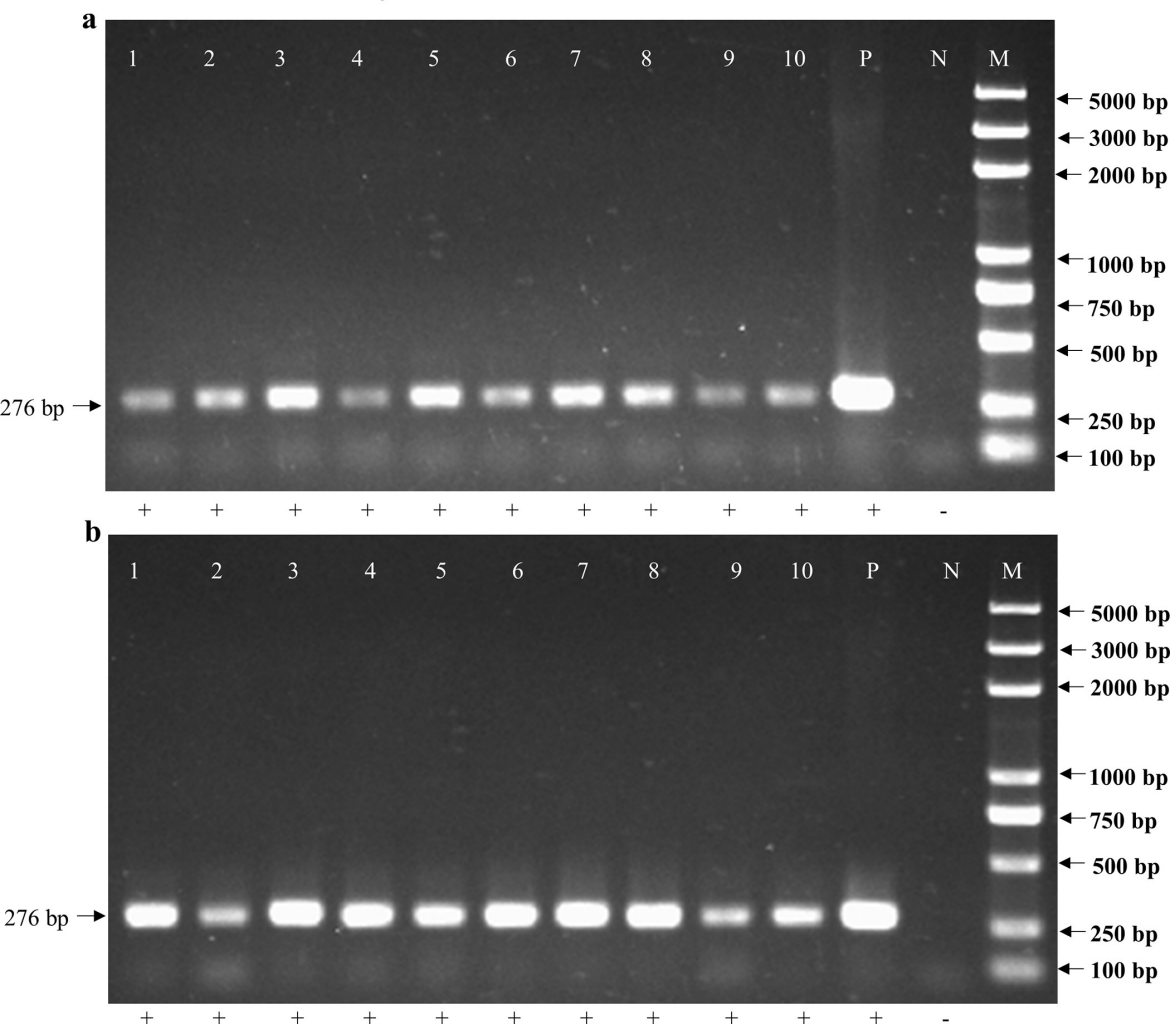
Amplification of *MsCHSB* gene fragments from *Mythimna separata* frass and exuviae. PCR products (276 bp) from DNA extracted from frass (a) and exuviae (b) separated on 1.5% agarose gel. Lanes 1–10 = Samples 1 to 10; P = positive control; N = negative control; M = molecular weight marker (5000 bp ladder) (Takara). *MsCHSB* fragments are indicated by arrows on the left. +, with indicated band; -, without indicated band.

**Fig 2:**
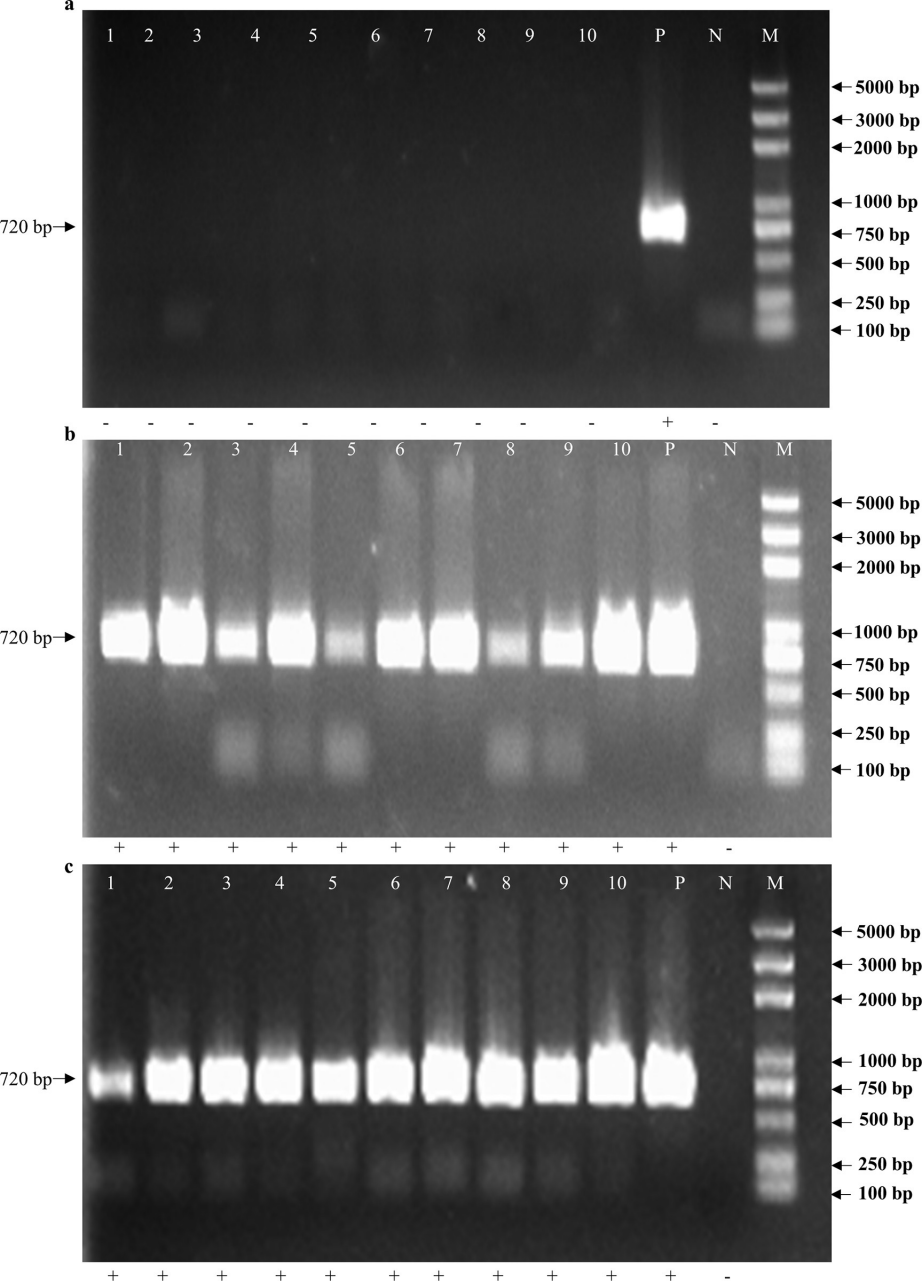
Amplification of *COI* gene fragments from *Mythimna separata* frass and exuviae. PCR products (720 bp) from DNA extracted from frass (a) and exuviae (b), and frass after Multiple Displacement Amplification (c) separated on 1% agarose gel. Lanes 1–10 = Samples 1 to 10; P = positive control; N = negative control; M = molecular weight marker (5000 bp ladder) (Takara). *COI* fragments are indicated by arrows on the left. +, with indicated band; -, without indicated band.

For the feasibility of non-destructive testing at the laboratory scale, mature *M*. *separata* larvae (10) were used. These were reared in a 12-well plate containing the required food. Frass and exuviae were obtained from each individual. After collecting the frass and exuviae, DNA extraction was carried out. The DNA obtained from frass and exuviae was of sufficient quality for PCR amplification. PCR was used to amplify fragments of alleles for two endogenous loci, namely the chitin synthesis B (*MsCHSB*) gene, a 276 bp nuclear DNA fragment, and cytochrome oxidase C (*COI*) gene, a 720 bp mitochondrial DNA (mtDNA) fragment. To amplify the *MsCHSB* fragment from frass samples, PCR was carried out with an annealing temperature of 55°C, and thereby always obtained a *MsCHSB* amplicon band from frass and exuviae DNA samples (Fig 1). A mtDNA *COI* amplicon was obtained from all exuviae DNA samples when an annealing temperature of 58°C was used. However, these same conditions did not amplify the *COI* fragment from frass samples (Fig 2A and 2B). The reason for the failure of amplification from frass samples may have been too little target DNA due to degradation by nucleases, or because of PCR inhibitors. To overcome the limitations of small sample size and extremely low DNA concentrations, Multiple Displacement Amplification (MDA) technology was developed [18]. The MDA method was used to improve the detection threshold of DNA extracted from frass samples. After MDA, PCR of the *COI* fragment was done using an annealing temperature of 58°C, and amplification was achieved for all frass samples (Fig 2C). The sequences of amplified fragments from frass and exuviae were confirmed to be identical to those of the adult individual that generated the sample. This analysis demonstrated that large gene fragments from *COI* (720 bp) were amplified from *M*. *separata* frass and exuviae.

**Fig 3:**
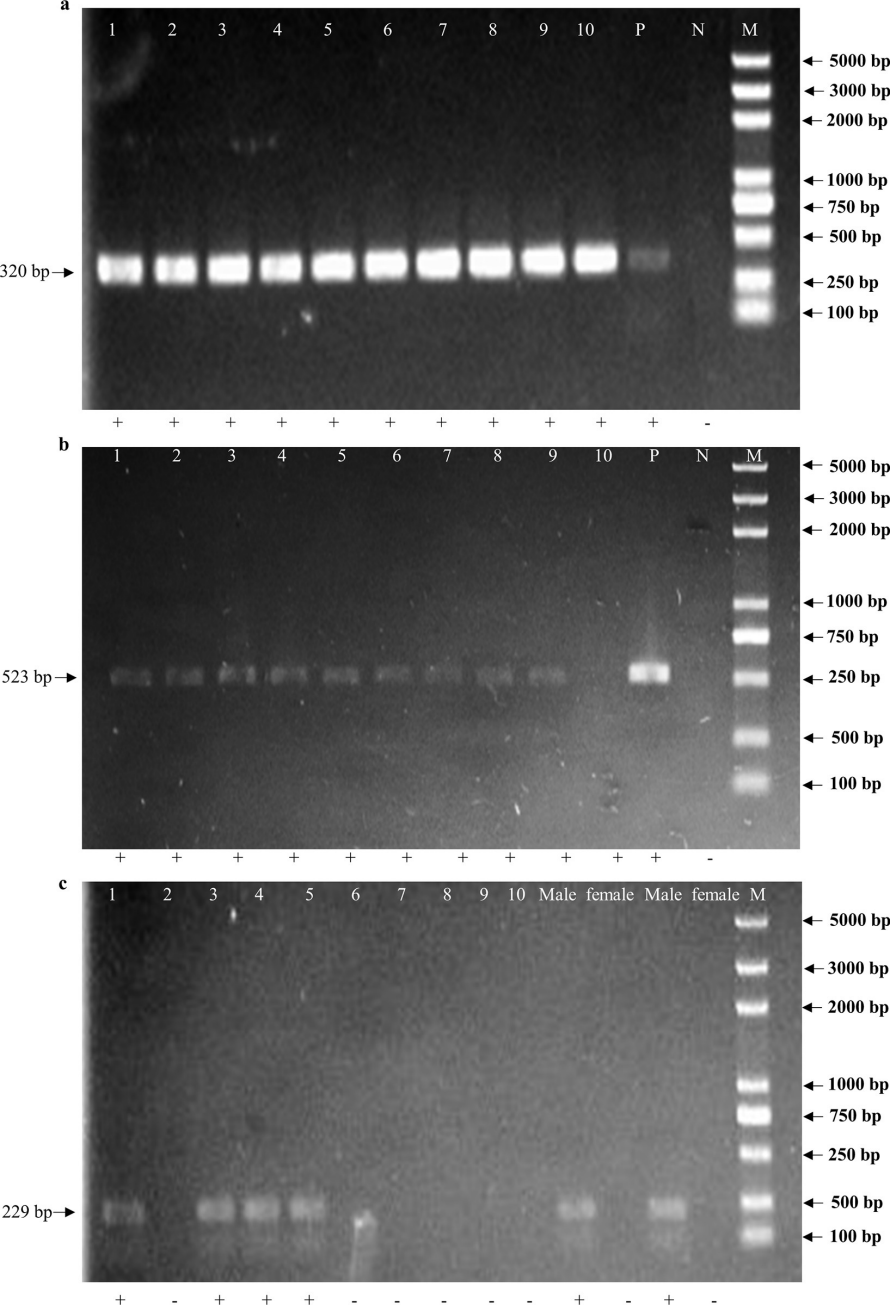
Amplification of endogenous genes *sryα*, *kl-5* and transgene *int* fragments from transgenic *Drosophila melanogaster* waste. PCR products of *sryα* (320 bp) (a), *int* (523 bp) (b) and *kl-5* (229 bp) (c) from DNA extracted from waste and separated on 1% agarose gel. Lanes 1–10 = samples 1 to 10; Male = male adult; Female = female adult; M = molecular weight marker (5000 bp ladder) (Takara). The *sryα* (a), *int* (b) and *kl-5* (c) fragments are indicated by arrows on the left. +, with indicated band; -, without indicated band.

*Drosophila melanogaster* is recognized as a model organism for the studies of developmental biology, genetics, and ecology [19]. Although many transgenic fly lines have been developed, there is no easy way to characterize these, or to differentiate males from females, at an early larval stage. To determine whether the non-destructive approach used here could also be applied to *D*. *melanogaster*, we obtained second instar stage larvae (Bloomington stock number: 24865) and reared them in 1.5 ml sterile microcentrifuge tubes with a small amount of food. After three days, the food was collected along with insect waste, and used for DNA extraction. We amplified fragments from three different loci. There were two endogenous genes, the *serendipity α* (*sryα*) locus [20], the male fertility locus *kl-5* [21], and one transgene, the ΦC31 integrase (*int*). Amplification of the endogenous *sryα* fragment was successful, and all samples gave the expected 320 bp band (Fig 3A). To amplify the transgene ΦC31 *int* fragment of 523 bp, DNA was extracted from 10 food samples of transgenic *D*. *melanogaster* larvae. Out of the ten samples tested, nine gave an amplification product (Fig 3B). This result confirms that transgene DNA can be detected as efficiently as endogenous genes by this method. The *kl-5* gene is a Y chromosome-specific gene, so it can be used as a molecular marker for maleness. This was confirmed by amplifying a 229 bp *kl-5* fragment with DNA prepared from whole bodies of two pairs of female and male fruit fly adults (Fig 3C). Feasibility of sex identification was confirmed at the early larval stage. From ten food samples (randomly selected), a *kl-5* fragment was detected in four of these (Fig 3C), therefore, the male to female ratio was close to the expected 1:1. Endogenous and exogenous genes were successfully amplified from waste food samples, and our results confirmed that this non-destructive approach could be very useful for identifying *D*. *melanogaster* transgenic lines, and determining sex in very early larval stages.

## Discussion

In the present study, a universal, simple and sensitive method was developed for non-invasive, non-lethal genotyping of a large insect, *M*. *separata*, and a small insect, *D*. *melanogaster*, by amplification of endogenous genes and transgenes located in the nuclear and mitochondrial genomes. DNA from *M*. *separata* frass and exuviae, and from *D*. *melanogaster* food waste was successfully extracted using a standard technique, and in a relatively short period of time. Sufficient DNA was extracted to allow PCR amplification of short size DNA fragments from most samples. The simplicity of this approach means that it can be developed into a high-throughput procedure. Non-destructive and non-lethal sampling of insects using this technique will allow us to collect DNA for population structure studies, parentage analyses, and to assess fitness.

DNA was extracted from fresh frass and exuviae samples from larvae of *M*. *separata* reared in individual wells of a 12-well plate. Exuviae are relatively fragile and persist for only a few days, so sample age may account for some PCR failures reported by others [11]. For this reason, we collected exuviae at a very early stage, stored them for a period, and then extracted DNA. The ages of some samples might account for PCR failure or for very weak bands. Nevertheless, short PCR products were successfully obtained from both frass and exuviae samples. Frass was an unreliable source of DNA for amplification of the larger mitochondrial gene *COI*, although amplification of this fragment from exuviae was successful. The *COI* DNA concentration in frass may have been below the threshold for successful PCR because frass contains relatively high levels of nucleases. To overcome these problems, pre-amplification of DNA by MDA has been shown to increase sensitivity of PCR. Therefore, the MDA method was used to improve the detection threshold from frass samples. PCR after MDA allowed successful amplification of *COI* from the frass (Fig 2C). This suggests that problematic samples of other species should respond to this technique, notably field samples. It is important to note that most DNA degradation happens when enzyme activation occurs at hydration, or when they are exposed to sunlight (UV-damage), so rapid collection and analysis of exuviae and frass is recommended.

The model insect species *D*. *melanogaster* has been widely used to construct transgenic lines for basic and applied research. Complex genetic manipulations, such as Flp/FRT and Cre/Loxp-mediated recombination [17], ZFN, TALEN, and CRISPR/Cas9-mediated genome editing are possible in this organism [16]. Although marker genes are used to track these manipulations, genotyping of larvae at an early developmental stage may also facilitate tracking transgenic insects. Currently, sampling methods for DNA preparation in *D*. *melanogaster* involve excision of adult body parts [22] because first and second instar stage larvae are too small to excise body parts or to obtain hemolymph for genotyping without affecting later development and reproduction. We applied the approach used for *M*. *separata* to second instar *D*. *melanogaster* larvae reared individually in 1.5 ml sterile microcentrifuge tubes. After three days, the remaining food left in the tube was collected for DNA extraction. Microcentrifuge tubes were easy to handle and it was simple to recover food without damage to the larva. The only requirements were to rear the larvae individually and then collect the food separately. This method may, therefore, allow high-throughput screening of individual insects raised this way.

The use of transgenic insects for release into natural populations is considered an environmentally-friendly technique for control of some pest species, and it is increasingly employed around the world [23]. Occasionally, transgenic insects may escape from laboratories. The external environment is different to that of the laboratory, and so laboratory-reared insects may behave differently when released. It will be necessary to monitor the relationship between genotypes and behavior, physiology, and reproduction of these released or escaped transgenic insects. The method presented here offers a simple and straightforward approach to this kind of monitoring, due to its simple, non-destructive, and high-throughput characteristics.

The identification of sex in insect adults and pupae is relatively easy in some species due to morphological appearance and presence of external genital organs. However, insect sex determination at the larval stage is usually more difficult. Many insect species show sex differences in development, physiology, and behavior, even in larval stages. Therefore, it is necessary to determine the sex of observed individuals in some studies, especially for ethological studies. Our non-invasive, non-lethal method easily identified males by PCR amplification of the sex-limited gene *kl-5*. PCR did not always succeed when using DNA obtained directly from frass, exuviae, or other waste products, which may result in a low rate of misidentification of sex. Other Y chromosome specific loci, such as other *KL* genes, increase the likelihood of correct identification. Addition of an MDA step before PCR for *kl-5*, as used for *COI* detection from *M*. *separata* frass, is an option not explored here. If this simple approach is successful, it will greatly facilitate the research to identify gender in insects that cannot be differentiated morphologically.

In this study, larvae were reared for three days with food to collect enough waste food material for DNA preparation. This approach yielded robust amplification bands (Fig 3C). In future, it may be possible to collect DNA and do genotyping after a shorter rearing time, and this may be particularly feasible if the MDA technique is also applied.

We obtained positive results using inexpensive, simple, and sensitive DNA extraction and amplification methods. More experiments using improved extraction techniques, e.g. column-based techniques, might increase both the quality and quantity of template DNA from insect samples obtained using non-invasive and non-lethal methods [24]. Such advances will increase the reliability of DNA extraction and allow amplification of other molecular markers.

## Materials and methods

The two DNA extraction experiments were carried out using individuals of *M*. *separata* and *D*. *melanogaster*. In the first experiment, third instar larvae of *M*. *separata* (originally collected from a field in Wuhan) were reared in a DNA-free 12-well plate with 0.5 g artificial diet with slight modifications as adopted by Ganbaatar *et al*. [25]. The rearing plate was cleaned with bleach to ensure that it was not contaminated with DNA from other individuals. After 24 h, frass was collected from each well into a 1.5 ml microcentrifuge tube. Exuviae were collected every 2–3 days. The experiment was continued for up to six days. Exuviae were then transferred into fresh, sterile microcentrifuge tubes and stored at –80ºC until use. For *D*. *melanogaster*, flies were obtained from the Core Facility of Drosophila Resource and Technology, SIBCB, CAS (Bloomington stock number: 24865, genotype: y [1] M {vas-int. Dm} ZH-2A w [*]; PBac{y[+]-attP-3b} VK00016). Second instar larvae were reared individually in 1.5 ml sterile microcentrifuge tubes with 0.15 g standard artificial diet [26]. After 3–4 days, larvae were removed carefully, food waste containing fecal material was collected from each larva separately and used for DNA extraction.

DNA was extracted from *M*. *separata* frass, exuviae, and from food waste using a standard method [27]. In brief, frass, exuviae and food samples were added to 2 ml DNA lysis buffer (50 mM Tris, 500 mM NaCl, 10 mM EDTA and 0.2% SDS, pH 8.0) and ground thoroughly using sterilized Dounce tissue grinders. The samples were centrifuged at 13,000 rcf for 5 min. The supernatant was transferred to fresh microcentrifuge tubes and 10 μl proteinase K (Takara) was added followed by incubation at 55°C for 2 h. After incubation, 100 μl sodium acetate (NaAc) was added and mixed gently. After this, phenol (550 μl) was added and mixed gently for 5–10 min. Samples were centrifuged at 13,000 rcf for 5 min, and the upper (aqueous) phase was transferred to new tubes before the phenol extraction was repeated. Then, two volumes of cold absolute ethanol was added, mixed, and DNA was pelleted at 12,000 rpm for 10–15 min. A final wash was done with 1 ml 70% ethanol. The DNA pellet was air-dried and dissolved with 40 μl TE buffer. DNA concentration was calculated from optical density at 260/280 nm.

The reaction mixture (10 μl final volume) for PCR contained 2 μl 5× PrimeSTAR plus Buffer (Takara), 0.8 μl dNTP mix (2.5 μM), 0.1 μl PrimeSTAR HS DNA Polymerase (0.5 U, Takara), 0.2 μl of each primer (10 μM), and DNA template (~50 ng). All the primers for PCR are listed in Table 1. All amplifications were performed using the following conditions unless otherwise stated: 95°C for 4 min to activate the PrimeSTAR DNA polymerase, followed by 35 cycles at 98°C for 10 s, 55°C for 10 s, and 72°C for 30 s, followed by a final extension step of 10 min at 72°C. The nuclear DNA fragment of *M*. *separata* chitin synthesis B gene (*MsCHSB*, 276 bp) and mitochondrial DNA fragment of Cytochrome oxidase C subunit (*COI*, 720 bp) [28], were amplified directly from *M*. *separata* exuviae DNA. For *COI* and the male fertility locus (*kl-5*, 229 bp) amplification, annealing temperatures of 58°C and 50°C were used respectively.

**Table 1:** Primers used for PCR amplification and sequencing of *Mythimna separata* and *Drosophila melanogaster* genes in this study.

Target gene	Forward Sequence (5’-3’)	Reverse Sequence (5’-3’)	Tm(°C)
*MsCHSB*^a^	TTTGCTGCCTGTAAAACTGCC	GTTCGATAAAATGCACACGGAT	55
*COI*^*b*^	GGTCAACAAATCATAAAGATATTGG	TAAACTTCAGGGTGACCAAAAAATCA	58
*sry α*^c^	AGGACTAGTCCGCGGTTTGCTAGATTAATCTAAGAAGCCC	AACAAGCTTAAGGCTGTTCTATCAGATGTGCT	55
*kl-5*^*d*^	TAGCGCTCTTCAGACTAATC	GTCTCTATTCTGGTAGTGAT	50
*Int*^e^	GGAAACGTCATGGACCTGAT	CGTCCGGCTTCTTCTTGTAG	55

^a^ Chitin synthesis B gene

^b^ cytochrome oxidase C

^c^serendipity α gene

^d^ male fertility factor

^e^ΦC31 integrase gene.

The *COI* region from *M*. *separata* frass DNA was amplified from all samples using Multiple Displacement Amplification (MDA), a non-PCR-based DNA amplification technique that can rapidly amplify minute amounts of DNA samples to a reasonable quantity by using the high fidelity, processivity and standard displacement capacity of phi 29 DNA polymerase [18]. The MDA reaction was carried out in a 20 μl final volume containing 2 μl 10× reaction buffer with 1.6 μl dNTP mix (2.5 μM each), 0.5 μl phi29 DNA polymerase (0.5 U, New England Biolabs), 0.5 μl hexamer primer (10 μM), and DNA template (~50 ng). These samples were incubated at 30°C for 3–4 h followed by deactivation of the enzymes by heating to 65°C for 12 min. After deactivation, the product was purified through an AxyGen PCR purification kit, diluted finally with 20 μl eluent water and stored at -20°C for future use.

The amplified products were separated on 1% or 2% agarose gels in 1x TAE buffer, and the bands were recorded using a gel documentation system. The DNA from exuviae and frass of 10 individuals that gave successful amplification of *COI* was sequenced. Amplification and analysis were performed in duplicates for all samples.

## Supporting information

S1 Sequence This is the sequencing results for cytochrome oxidase C (*COI*) gene. (RAR)
